# Cutaneous metastasis of unknown primary presenting as massive and invasive abdominal lesion: an elective approach*

**DOI:** 10.1590/abd1806-4841.20153793

**Published:** 2015

**Authors:** Paolo Lido, Giovanni Paolino, Andrea Feliziani, Letizia Santurro, Mauro Montuori, Flavio de Sanctis, Piero Rossi, Giuseppe Petrella, Edoardo Ricciardi, Giuseppe Fusano, Orlandi Augusto, Patrizio Polisca

**Affiliations:** 1 Università degli Studi di Roma Tor Vergata – Roma, Italia.; 2 Università degli Studi di Roma “La Sapienza” - Roma, Italia.

**Keywords:** Electrochemotherapy, Neoplasm metastasis, Neoplasms, unknown primary

## Abstract

We describe herein what is to our knowledge the first reported case of an invasive cutaneous metastasis with unknown primary, electively treated solely with electrochemotherapy. We describe a female patient with a large, invasive and painful lesion in her hypogastric region, extending up to the pubic area. The cutaneous biopsy and instrumental and laboratory analyses, all failed to reveal the primary site. A final diagnosis of cutaneous metastasis with unknown primary was made and treatment was performed with electrochemotherapy. Our case highlights the importance of interdisciplinary choices in clinical practice to cope with the lack of a primary site and to improve quality of life, since no standardized therapy exists for these classes of patients.

## INTRODUCTION

Among cutaneous metastases, those with unknown primary (CMUP), account for 4.4% of all cases.^1,2^ Recent reports have found that in cases of metastatic malignancies of unknown primary origin, primary sites are identifiable in only 20-25% of cases before death.^2^

Since the average survival time following the appearance of a cutaneous metastasis is 3-7.5 months, and because it sometimes presents as invasive lesions, an appropriate diagnosis is needed to identify patients with treatable disease and those with a more favorable prognosis.^2,3^

We describe a CMUP of the abdominal region, reporting the relative diagnostic procedures and management involved. Given the impossibility of wide surgical excision (and/or systemic chemotherapy) and due to the unusual histotype, we opted for electrochemotherapy (ECT). ECT is a local treatment based on the phenomenon of electroporation, characterized by the formation of pores (by applying an electric field) in the cell-membrane, thus making it permeable and enabling the passage of anti-neoplastic agents (such as bleomycin or cis-platin) in the cytosol, resulting in a cytotoxic process.^4^

## CASE REPORT

**Figure 1:**
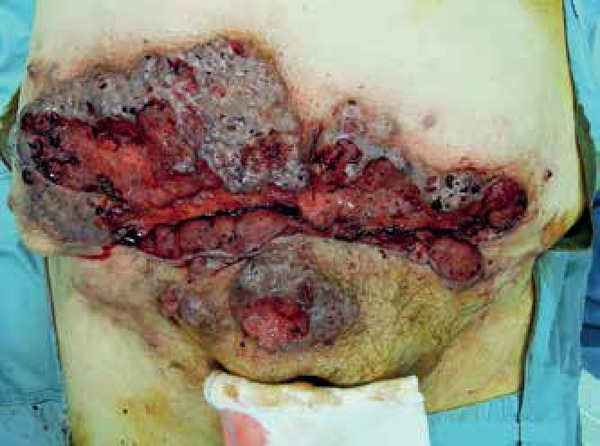
Large and painful lesion in the hypogastric region, extending up to the pubic and supra pubic area. The lesion appeared ulcerated and bleeding

A 54-year-old Caucasian woman presented to our department with a 6-month history of a large and painful lesion on her hypogastric region, extending up to the pubic area. The lesion was ulcerated, malodorous, bleeding, and ranged 15cm X 9cm in diameter. At the periphery of the lesion, several nodulations were visible (Figure 1).

The patient's past medical history was significant for HCV infection, tonsillectomy and a cholecystectomy (treatment for gallstones). The physical examination was negative for other noteworthy clinical signs.

**Figure 2:**
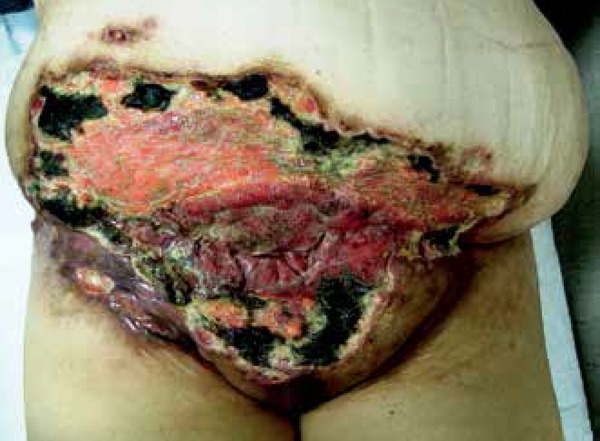
A. Dermal proliferation, constituted by an infiltration of undifferentiated adenocarcinoma with focal aspects of clear cells. (10 X, hematoxylin-eosin). B: Tumor cells positive for CK-7(10 X)

The histological examination of the incisional biopsy showed a dermal proliferation, constituted by an infiltration of a poorly differentiated adenocarcinoma, characterized by the presence of focal areas with psammomatous aspects and clear cells (Figure 2A). Immunohistochemical studies showed that the tumor cells were reactive for cytokeratin (CK) 7 and CK 5/6, while they were negative for CD10, CK 20 and WT1 (Figure 2B). According to the biopsy, an esophagus-gastro-duodenoscopy, a colonoscopy, trans-vaginal ultrasound, bilateral mammography and bronchoscopy, were performed without evidence of the primary malignancy. The computed tomography and the total-body positron emission computed tomography revealed that the radiotracer was established in the abdominal region, but without highlighting the primary site. The routine laboratory investigations and dosages of the oncologic markers (including AFP, β-HCG and CA125) did not show significant alterations.

Based on the patient's history, the cutaneous biopsy and instrumental analyses, a final diagnosis of CMUP was made.

**Figure 3:**
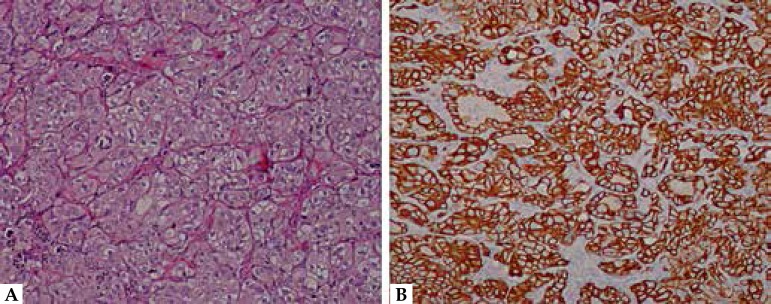
Tumor lesion after about 60 days of electrochemotherapy. Complete response in the peripheral and smaller nodules, with a partial response in the biggest ones

Given the extent of the skin lesions, the clinical features (pain, bleeding and smelliness), the fact that oncological valuation did not present indications for systemic chemotherapy, and following the patient's personal opinion, we performed electrochemotherapy. The patient received intravenous bleomycin (at a dosage of 26.5 mg) under general anesthesia, followed (8 minutes later) by electroporation on the tumoral lesion through the pulse generator Cliniporator^TM^ (IGEA S.p.A, Carpi, Italy). A hexagonal array of electrodes was used during this procedure. Subsequently, we arranged weekly clinical follow-ups. At 60 days, we observed a complete response in peripheral and smaller nodules, with a partial response in the biggest ones (over 3cm in size) (Figure 3). Pain and bleeding were significantly reduced.

## DISCUSSION

The patient's age, along with the cutaneous lesion's adenocarcinoma histotype, corresponded to the clinical-pathological features of CMUP, most of which are reported in the literature (2, 5). However, undifferentiated histotypes in the CMUP spectrum are described in only 30% of these cases.^2,5^

Based on the anatomical region involved and the histopathological features, the main differential diagnoses included cutaneous metastasis of an ovarian adenocarcinoma and a primitive papillary serous neoplasia of the peritoneum (PPSC).^3^

Cutaneous metastases occur in 3.5-4% of patients with ovarian carcinoma and tend to appear late in the course of the disease.^3^ Clinically, they are represented by multiple small nodules, herpetiform erythematous lesions and/or scarring plaques, often affecting the lower abdomen and anterior chest. Histologically, cutaneous metastases from ovarian cancer reproduce a well-differentiated adenocarcinoma; while upon immunohistochemistry they show positivity to CK7, CA125, and negativity to CK20. However, in this case the instrumental negativity for a primary ovarian carcinoma, associated with negativity to CA125, had determined a valid discrimination in the diagnosis.^3^ PPSC is a rare tumor of the peritoneum, clinically similar to an epithelial ovarian carcinoma, entailing a similar management approach and overall survival rate. Currently, these two entities are histologically indistinguishable and about 10% of epithelial ovarian cancers are reclassified as PPSC.^6^ Age, gender, CK7-positivity and the anatomical site affected were linked to a diagnosis of PPSC. Nevertheless, the low levels of CA125 and negativity to WT1 deviated from a PPSC diagnosis. The primary site of our cutaneous lesion remained unknown.

Electrochemotherapy (ECT) has proven effective in treating several types of neoplasia and guaranteeing an improvement in overall quality of life.^7^ It combines chemotherapy and electroporation to increase drug uptake into cancer cells. Further, in addition to the well-established, direct cytotoxic effect on tumor cells, ECT has an indirect vascular disrupting action, resulting in extensive tumor cell necrosis, leading to complete regression of tumors.^8^ It is an excellent option for patients suffering from cutaneous metastasis where other treatments have failed or cannot be applied.^9^ In our case, ECT proved to be a valid palliative treatment, improving quality of life (reducing pain, smelliness and bleeding), which is unfortunately very low among these patients.^10^

In conclusion, reliable clinical information from careful morphological and instrumental evaluation should help identify the cause of most CMUP cases, while immunohistochemistry should be considered mainly in the rare instances of poorly differentiated CMUP.^1^ The absence of a standardized protocol for this class of patients and the poor prognosis of adenocarcinoma in setting of unknown primary, require a multidisciplinary approach. The reductions in bleeding and pain, together with better adherence to ECT treatment by our patient, were the main focal points of this case report.
